# A new kind of inner superefficient points

**DOI:** 10.1186/s13660-017-1452-6

**Published:** 2017-08-01

**Authors:** Yihong Xu, Lei Wang, Chunhui Shao

**Affiliations:** Department of Mathematics, Nanchang University, Nanchang, 330031 China

**Keywords:** 90C59, inner superefficient point, superefficient point, near cone-subconvexlikeness

## Abstract

In this paper, some properties of the interior of positive dual cones are discussed. With the help of dilating cones, a new notion of inner superefficient points for a set is introduced. Under the assumption of near cone-subconvexlikeness, by applying the separation theorem for convex sets, the relationship between inner superefficient points and superefficient points is established. Compared to other approximate points in the literature, inner superefficient points in this paper are really ‘approximate’.

## Introduction

The approximate efficient solution is an important notion of vector optimization theory. Many interesting results have been obtained in recent years. For example, Loridan [1, 2] introduced the concept of *ϵ*-solutions in general vector optimization problems. Rong and Wu [3] considered cone-subconvexlike vector optimization problems with set-valued maps in general spaces and derived scalarization results, *ϵ*-saddle point theorems, and *ϵ*-duality assertions using *ϵ*-Lagrangian multipliers. Qiu and Yang [4] studied the approximate solutions for vector optimization problem with set-valued functions, and derived the scalar characterization without imposing any convexity assumption on the objective functions. The authors of [5] introduced the notion of *ϵ*-strictly efficient solution for vector optimization with set-valued maps. Under the assumption of the ic-cone-convexlikeness for set-valued maps, the scalarization theorem, *ϵ*-Lagrangian multiplier theorem, *ϵ*-saddle point theorems and *ϵ*-duality assertions were established for *ϵ*-strictly efficient solution. The concept of nondominated solutions with variable domination structures or variable orderings was introduced by Yu [6]. This is a generalization of the nondominated solution concept with fixed domination structure in multicriteria decision making problems. Chen [7] introduced a nonlinear scalarization function for a variable domination structure,and by applying this nonlinear function characterized the weakly nondominated solution of multicriteria decision making problems.

On the other hand, proper efficiency is a natural concept in vector optimization. Borwein [8] introduced a new kind of proper efficiency,namely super efficiency. Super efficiency refines the notions of efficiency and other kinds of proper efficiency. Fu [9] and Zheng [10] gave two different generalizations of super efficiency in a locally convex space. Xu and Zhu [11] investigated the set-valued optimization problem with constraints in the sense of super efficiency in locally convex linear topological spaces. Hu and Ling [12] studied the connectedness of the cone superefficient point set in locally convex topological vector spaces. In [3, 4, 11–14], the nonemptiness of the interior of the order cone must be satisfied when proving optimization results.

However, in many cases, the ordering cone has an empty interior. For example, for each $1< p<+\infty$, the normed space $l_{p}$ partially ordered by the positive cone is an important space in applications; the positive cone has an empty interior. Another example is the case when *C* is the Cartesian product $C'\times C''$ of a trivial cone $C'=\{0\}$ and a cone $C''$ having a nonempty interior. Thus, to study the vector optimization problem under the condition that ordering cone has an empty interior has become an important topic [15, 16].

Let *Y* be a locally convex topological vector space; let $C\subset Y$ be a pointed closed convex cone and *B* be a base of *C*. Let $A\subset Y$ be nonempty.

Rong and Wu [3] showed that

1.1 $$ W\operatorname{Min}(A,C)\subset\epsilon-W\operatorname{Min}(A,C),\quad \forall \epsilon\in C. $$

The authors of [5] demonstrated that

1.2 $$ FE(A,B)\subset\epsilon-F\min(A,B), $$

and the inverse inclusion does not hold in general.

Naturally, we come up with the problem how to introduce the notion of approximate point and under what condition the set of inner points equals the set of primal points. When order cone *C* has an empty interior, how to investigate superefficient points?

Motivated by Yu [6], Zheng [10] and Gong [15], we will introduce a new kind of superefficient point (see Definition 2.2), under certain conditions, we will obtain the relationship between inner superefficient point and superefficient point (see Theorem 2.3). In the literature [1–5], approximate points were defined by adding *ϵ* or −*ϵ* to a set *A*, in this paper, inner superefficient points will be introduced by a variable domination structure.

## Notation and preliminaries

In the remainder of the paper, let *Y* be a normable locally convex topological vector space; $C\subset Y$ be a pointed closed convex cone; $Y^{\ast}$ be the dual space of *Y*. The positive dual cone $C^{\ast}$ of *C* is denoted by

$$C^{\ast}=\bigl\{ f\in Y^{\ast}: f(c)\geq0, \forall c\in C\bigr\} . $$

For a set $B\subset C$, we write

$$\operatorname{cone}B = \{\lambda b: \lambda\geq0, b \in B\}. $$

The closure of set *B* is denoted by cl*B*. A convex subset *B* of a cone *C* is a base of *C* if $0{\notin }\operatorname{cl}B$ and $C=\operatorname{cone}B$.

Let *B* be a base of *C*, then $0\notin\operatorname{cl}B$. By the separation theorem, there exists $\bar{f}\in Y^{\ast}\backslash\{0\}$ such that

2.1 $$ r=\operatorname{inf}\bigl\{ \bar{f}(b): b\in B\bigr\} > 0. $$

Let

2.2 $$ V_{B}=\biggl\{ y\in Y: \bigl\vert \bar{f}(y) \bigr\vert < \frac{r}{2}\biggr\} . $$

Define the neighborhood family of 0 in *Y* as follows:

2.3 $$ N(0)=\{V\subset V_{B}: V \mbox{ is an open convex balanced bounded neighborhood of }0\}. $$

All bounded subsets of *Y* are denoted by Ω, and for any $M\in\Omega$, let

$$P_{M}(f)=\operatorname{sup}\bigl\{ \bigl\vert f(y) \bigr\vert :y \in M\bigr\} ,\quad f\in Y^{\ast}. $$

Let $\tau^{\ast}$ denote the locally convex topology induced by the quasi-norm family $\{P_{M}:M\in\Omega\}$ on $Y^{\ast}$, $\operatorname{int} C^{\ast}$ denote the interior set of $C^{\ast}$ in the sense of $\tau^{\ast}$.

### Theorem 2.1

*Let* *B* *be a bounded base of* *C*. *Then*

2.4 $$ \operatorname{int}C^{\ast}=\bigcup_{U\in N(0)} \operatorname {int}\bigl(\operatorname{cl}\,\operatorname{cone}(B+U) \bigr)^{\ast}. $$

### Proof

Since $C\subset\operatorname{cl}\,\operatorname {cone}B\subset\operatorname{cl}\,\operatorname{cone}(B+U)$, for any $U\in N(0)$. we get $(\operatorname{cl}\,\operatorname{cone}(B+U))^{\ast}\subset C^{\ast}$, so $\operatorname{int}(\operatorname{cl}\,\operatorname {cone}(B+U))^{\ast}\subset\operatorname{int}C^{\ast}$. Thus

$$\bigcup_{U\in N(0)}\operatorname{int}\bigl( \operatorname{cl}\,\operatorname {cone}(B+U)\bigr)^{\ast}\subset \operatorname{int}C^{\ast}. $$

In what follows, we prove

$$\operatorname{int}C^{\ast}\subset\bigcup_{U\in N(0)} \operatorname {int}\bigl(\operatorname{cl}\,\operatorname{cone}(B+U) \bigr)^{\ast}. $$

Take $f_{0}\in\operatorname{int}C^{\ast}$, then there exists $\epsilon _{0}\in(0,1)$ such that

$$f_{0}-\epsilon_{0}\bar{f}\in C^{\ast}, $$

it follows from $B\subset C$ that

$$(f_{0}-\epsilon_{0}\bar{f}) (b)\geq0,\quad \mbox{for any } b \in B, $$

which implies, together with (2.1), that

2.5 $$ f_{0}(b)\geq\epsilon_{0}\bar{f}(b)\geq\epsilon_{0}r, \quad \mbox{for any } b\in B. $$

Take $M_{0}\in\Omega$, let

2.6 $$ U_{0}=\biggl\{ y\in Y : \bigl\vert f_{0}(y) \bigr\vert < \frac{\epsilon_{0}r}{2}\biggr\} \cap M_{0}, $$

then $U_{0}\in N(0)$. Denote

2.7 $$ V^{\ast}=\biggl\{ f\in Y^{\ast}: \operatorname{sup}\bigl\{ \bigl\vert f(t) \bigr\vert : t\in B+U_{0}\bigr\} < \frac{\epsilon_{0}r}{4}\biggr\} . $$

Since *B* and $U_{0}$ are bounded, we conclude that $V^{\ast}$ is a neighborhood of 0 in $Y^{\ast}$.

In the following, we need to prove that

$$f_{0}+V^{\ast}\subset \bigl(\operatorname{cl}\,\operatorname {cone}(B+U_{0})\bigr)^{\ast}, $$

that is,

$$f_{0}+f\in \bigl(\operatorname{cl}\,\operatorname{cone}(B+U_{0}) \bigr)^{\ast },\quad \mbox{for any }f\in V^{\ast}. $$

For each $t\in B+U_{0}$, there exist $b\in B$, $u\in U_{0}$ such that $t=b+u$, then,

$$(f_{0}+f) (t)=f_{0}(t)+f(t)=f_{0}(b)+f_{0}(u)+f(t). $$

Combining (2.5), (2.6) and (2.7), we see that

$$(f_{0}+f) (t)>\epsilon_{0}r-\frac{\epsilon_{0}r}{2}- \frac{\epsilon _{0}r}{4}=\frac{\epsilon_{0}r}{4}>0,\quad \forall t\in B+U_{0}. $$

For each $s\in\operatorname{cl}\,\operatorname{cone}(B+U_{0})$, there exist $\lambda_{n}\geq0$, $t_{n}\in B+U_{0}$, such that $s=\lim_{n\rightarrow\infty}\lambda_{n}t_{n}$, then

$$(f_{0}+f) (s)=\lim_{n\rightarrow\infty}(f_{0}+f) ( \lambda_{n}t_{n})=\lim_{n\rightarrow\infty} \lambda_{n}(f_{0}+f) (t_{n})\geq0. $$

Thus,

$$f_{0}+f\in \bigl(\operatorname{cl}\,\operatorname{cone}(B+U_{0}) \bigr)^{\ast}. $$

Hence,

$$f_{0}+V^{\ast}\subset \bigl(\operatorname{cl}\,\operatorname {cone}(B+U_{0})\bigr)^{\ast}. $$

Therefore,

$$f_{0}\in \operatorname{int}\bigl(\operatorname{cl}\,\operatorname {cone}(B+U_{0})\bigr)^{\ast}. $$

Consequently,

$$\operatorname{int}C^{\ast}\subset\operatorname{int}\bigl( \operatorname{cl}\,\operatorname{cone}(B+U_{0})\bigr)^{\ast}. $$

The proof is complete. □

### Definition 2.1

[10]

Let $A\subset Y$ be nonempty, $\bar {y}\in A$ is said to be a superefficient point of *A*, written as $\bar{y}\in \operatorname{SE}(A, C)$, if, for each neighborhood *V* of 0 in *Y*, there exists a neighborhood *W* of 0 such that

$$\operatorname{cone}\bigl(A-\{\bar{y}\}\bigr)\cap(W-C)\subset V. $$

Suppose $U\in N(0)$, let

$$C_{U}(B)=\operatorname{cl}\,\operatorname{cone}(B+U). $$

The notion $C_{U}(B)$ will be used throughout this paper.

In the following, with variable ordering, we introduce the concept of inner superefficient point.

### Definition 2.2

Let $A\subset Y$ be nonempty, $\bar{y}\in A$ is said to be an inner superefficient point, if $\bar{y}\in \operatorname{SE}(A, C_{U}(B))$, for some $U\in N(0)$. That is, for each neighborhood *V* of 0 in *Y*, there exists a neighborhood *W* of 0 such that

$$\operatorname{cone}\bigl(A-\{\bar{y}\}\bigr)\cap\bigl(W-C_{U}(B) \bigr)\subset V. $$

### Remark 2.1

It is clear that

$$\operatorname{SE}\bigl(A, C_{U}(B)\bigr)\subset \operatorname{SE}(A, C),\quad \forall U\in N(0). $$

In the following, we give the existence theorem of inner superefficient points.

### Theorem 2.2

*Let* *Y* *be a normable locally convex topological vector space*, *B* *be a bounded base of* *C*, *and* $A\subset Y$ *be a weakly compact set*. *Then for any* $U\in N(0)$, $\operatorname{SE}(A,C_{U}(B))\neq\emptyset$.

### Proof

Since *U* is bounded and *Y* is normable, in the same way as the proof of [8], Theorem 1.1, we can show that

$$\operatorname{cl}\,\operatorname{cone}(B+U)=\operatorname{cone}\,\operatorname{cl}(B+U). $$

Hence

$$C_{U}(B)=\operatorname{cone}\,\operatorname{cl}(B+U). $$

By (2.2) and (2.3), we conclude $\operatorname{cl}(B+U)$ is a bounded base of $C_{U}(B)$. From [10], Corollary 3.1, we deduce $\operatorname{SE}(A,C_{U}(B))\neq\emptyset$. □

Convexity plays a key role in optimization theory. Yang *et al.* [17] introduced a new class of generalized convexity termed near *C*-subconvexlikeness.

### Definition 2.3

[17]

Suppose $A\subset Y$ is nonempty, *A* is said to be nearly *C*-subconvexlike if $\operatorname{cl}\,\operatorname{cone}(A+C)$ is convex.

### Remark 2.2

Sach [16] introduced another convexity called ic-cone-convexlikeness. The authors of [18] obtain the following results:

1. when the ordering cone has nonempty interior, ic-cone-convexness is equivalent to near cone-subconvexlikeness;
2. when the ordering cone has empty interior, ic-cone-convexness implies near cone-subconvexlikeness, a counter example is given to show that the converse implication is not true.

Thus, near *C*-subconvexlikeness is a very generalized convexity.

### Theorem 2.3

*Suppose that for each* $y\in Y$, $A-\{\bar{y}\}$ *is nearly* *C*-*subconvexlike*, *and* *B* *is a bounded base of* *C*. *Then*

$$\bigcup_{U\in N(0)}\operatorname{SE}\bigl(A,C_{U}(B) \bigr)=\operatorname{SE}(A,C). $$

### Proof

Since $\operatorname{SE}(A, C_{U}(B))\subset \operatorname{SE}(A, C)$, $\forall U\in N(0)$,

$$\bigcup_{U\in N(0)}\operatorname{SE}\bigl(A,C_{U}(B) \bigr)\subset \operatorname{SE}(A,C). $$

In what follows, we prove

$$\operatorname{SE}(A,C)\subset\bigcup_{U\in N(0)} \operatorname{SE}\bigl(A,C_{U}(B)\bigr). $$

Take $\bar{y}\in \operatorname{SE}(A,C)$.

1^∘^ We show there exists $U\in N(0)$ such that

2.8 $$ \operatorname{cone}\bigl(A-\{\bar{y}\}\bigr)\cap(U-B)=\emptyset. $$

Indeed, since $0\notin\operatorname{cl}B$, there exists $V\in N(0)$ such that $-B\cap V=\emptyset$, hence,

2.9 $$ \frac{V}{2}\cap\biggl(\frac{V}{2}-B\biggr)=\emptyset. $$

As $\bar{y}\in \operatorname{SE}(A,C)$, for the above *V* there is a neighborhood *W* of 0 such that $\operatorname{cone}(A-\{\bar{y}\})\cap(W-C)\subset\frac{V}{2}$. Taking $U=W\cap\frac{V}{2}\cap V_{B}$, we get

2.10 $$ \operatorname{cone}\bigl(A-\{\bar{y}\}\bigr)\cap(U-C)\subset \frac{V}{2}. $$

By $B\subset C$, we conclude

$$\operatorname{cone}\bigl(A-\{\bar{y}\}\bigr)\cap(U-B)=\operatorname{cone} \bigl(A-\{ \bar{y}\}\bigr)\cap(U-C)\cap(U-B), $$

and from (2.10),

2.11 $$ \operatorname{cone}\bigl(A-\{\bar{y}\}\bigr)\cap(U-B)\subset\frac{V}{2} \cap (U-B). $$

Since $U\subset\frac{V}{2}$, it follows from (2.9) that $\frac{V}{2}\cap (U-B)=\emptyset$. In view of (2.11),

$$\operatorname{cone}\bigl(A-\{\bar{y}\}\bigr)\cap(U-B)=\emptyset. $$

2^∘^ In the following, we demonstrate

2.12 $$ \operatorname{cone}\bigl(A+C-\{\bar{y}\}\bigr)\cap(U-B)=\emptyset. $$

By contradiction, suppose that $\operatorname{cone}(A+C-\{\bar{y}\})\cap (U-B)\neq\emptyset$. Then there exist $\lambda_{1}\geq0$, $a_{1}\in A$, $c_{1}\in C$, $u_{1}\in U$, $b_{1}\in B$ such that

2.13 $$ \lambda_{1}(a_{1}+c_{1}-\bar{y})=u_{1}-b_{1}. $$

Since *B* is the base of *C*, there exist $\lambda_{2}\geq0, b_{2}\in B$ such that $c_{1}=\lambda_{2}b_{2}$, hence,

$$\lambda_{1}(a_{1}+\lambda_{2}b_{2}- \bar{y})=u_{1}-b_{1}. $$

Consequently,

$$\frac{\lambda_{1}}{1+\lambda_{1}\lambda_{2}}(a_{1}-\bar{y})=\frac {1}{1+\lambda_{1}\lambda_{2}}u_{1}- \biggl(\frac{1}{1+\lambda_{1}\lambda_{2}}b_{1}+\frac{\lambda_{1}\lambda _{2}}{1+\lambda_{1}\lambda_{2}}b_{2}\biggr). $$

Since *B* is convex, the above equal elements belong to $\operatorname {cone}(A-\{\bar{y}\})\cap(U-B)$, this is a contradiction.

Since *U* is open, it follows from (2.12) that

$$\operatorname{cl}\,\operatorname{cone}\bigl(A+C-\{\bar{y}\}\bigr)\cap (U-B)= \emptyset. $$

3^∘^ Since $A-\{\bar{y}\}$ is nearly *C*-subconvexlike, $\operatorname{cl}\,\operatorname{cone}(A+C-\{\bar{y}\})$ is convex. By the separation theorem of convex sets, there exists $\hat{f}\in Y^{\ast}\backslash\{0\}$ such that

2.14 $$ \operatorname{inf}\bigl\{ \hat{f}(k):k\in\operatorname{cl}\,\operatorname {cone} \bigl(A+C-\{\bar{y}\}\bigr)\bigr\} \geq\operatorname{sup}\bigl\{ \hat{f}(z):z\in U-B \bigr\} . $$

Since $0\in\operatorname{cl}\,\operatorname{cone}(A+C-\{\bar{y}\})$, thus,

$$\hat{f}(z)\leq0,\quad \forall z\in U-B. $$

Consequently,

$$\hat{f}(b)\geq\hat{f}(u), \quad \forall b\in B, u\in U. $$

Since *U* is absorbed, there exists $u_{0}\in U$ such that $\hat {f}(u_{0})>0$, thus,

2.15 $$ \hat{f}(b)\geq\hat{f}(u_{0})>0. $$

4^∘^ In what follows, we show

$$\hat{f}\in\operatorname{int}C^{\ast}. $$

Let

2.16 $$ V_{0}^{\ast}=\biggl\{ f\in Y^{\ast}:\operatorname{sup} \bigl\{ \bigl\vert f(b) \bigr\vert :b\in B\bigr\} < \frac {\hat{f}(u_{0})}{2}\biggr\} . $$

In the following, we show

$$\hat{f}+V_{0}^{\ast}\subset C^{\ast}. $$

In fact, for each $f\in V_{0}^{\ast}$, $c\in C$, there exist $\lambda\geq 0$, $b\in B$ such that $c=\lambda b$, then

$$(\hat{f}+f) (c)=\lambda\bigl(\hat{f}(b)+f(b)\bigr), $$

it follows from (2.15) and (2.16) that

$$(\hat{f}+f) (c)\geq\lambda\biggl(\hat{f}(u_{0})-\frac{\hat{f}(u_{0})}{2} \biggr)=\frac {\lambda}{2}\hat{f}(u_{0})\geq0. $$

Thus,

$$\hat{f}+f\in C^{\ast}. $$

That is

$$\hat{f}+V_{0}^{\ast}\subset C^{\ast}. $$

It means that

$$\hat{f}\in\operatorname{int}C^{\ast}. $$

5^∘^ From Theorem 2.1, there exists $\hat{U}\in N(0)$ such that

$$\hat{f}\in \operatorname{int}\bigl(\operatorname{cl}\,\operatorname {cone}(B+ \hat{U})\bigr)^{\ast}. $$

Thus there exists $\hat{\epsilon}\in(0,1)$ such that

2.17 $$ \hat{f}-\hat{\epsilon}\bar{f}\in \bigl(\operatorname{cl}\,\operatorname {cone}(B+\hat{U})\bigr)^{\ast}. $$

For each $b\in B$, $u\in\hat{U}\in N(0)$, it follows from (2.1)-(2.3) that

$$\bar{f}(b+u)=\bar{f}(b)+\bar{f}(u)>r-\frac{r}{2}=\frac{r}{2}. $$

Thus

$$\bar{f}(t)>\frac{r}{2},\quad \forall t\in B+\hat{U}. $$

From (2.17), we get

2.18 $$ \hat{f}(t)\geq\hat{\epsilon}\bar{f}(t)>\frac{r\hat{\epsilon}}{2}>0,\quad \forall t\in B+\hat{U}. $$

In what follows, we prove

$$\bar{y}\in \operatorname{SE}\bigl(A,C_{\hat{U}}(B)\bigr), $$

where $C_{\hat{U}}(B)=\operatorname{cl}\,\operatorname{cone}(B+\hat {U})$.

6^∘^ Since *Û* is bounded and *Y* is normable, in the same way as in the proof of [8], Theorem 1.1, we can show that

$$\operatorname{cl}\,\operatorname{cone}(B+\hat{U})=\operatorname{cone}\,\operatorname{cl}(B+\hat{U}). $$

Consequently,

2.19 $$ C_{\hat{U}}(B)=\operatorname{cone}\,\operatorname{cl}(B+ \hat{U}). $$

Since *B* is bounded and $\hat{U}\in N(0)$, we see that $\operatorname {cl}(B+\hat{U})$ is bounded. From (2.18), we get

2.20 $$ \operatorname{inf}\bigl\{ \hat{f}(t):t\in\operatorname{cl}(B+\hat{U})\bigr\} \geq \frac{r\hat{\epsilon}}{2}. $$

Thus,

$$0\notin\operatorname{cl}(B+\hat{U}). $$

Therefore, it follows from (2.19) that $\operatorname{cl}(B+\hat{U})$ is a bounded base of $C_{\hat{U}}(B)$. Let

2.21 $$ \tilde{U}=\biggl\{ y\in Y: \bigl\vert \hat{f}(y) \bigr\vert < \frac{r\hat{\epsilon}}{4}\biggr\} . $$

7^∘^ In the following, we prove

2.22 $$ \operatorname{cone}\bigl(A-\{\bar{y}\}\bigr)\cap\bigl[-\bigl(\tilde{U}+ \operatorname {cl}(B+\hat{U})\bigr)\bigr]=\emptyset. $$

By (2.14), since $\operatorname{cl}\,\operatorname{cone}(A+C-\{\bar{y}\} )$ is a cone and on which *f̂* has lower bound, we have

$$\hat{f}(k)\geq0,\quad \forall k\in\operatorname{cl}\,\operatorname {cone} \bigl(A+C-\{\bar{y}\}\bigr). $$

Since $0\in C$, we have $A-\{\bar{y}\}\in\operatorname{cl}\,\operatorname{cone}(A+C-\{ \bar{y}\})$, $\forall a\in A$, thus,

$$\hat{f}(a-\bar{y})\geq0. $$

We get

2.23 $$ \hat{f}(s)\geq0, \quad \forall s\in\operatorname{cone}\bigl(A-\{\bar{y}\} \bigr). $$

On the other hand, from (2.20) and (2.21), we see that

2.24 $$ \hat{f}(t)\geq\frac{r\hat{\epsilon}}{2}-\frac{r\hat{\epsilon}}{4}=\frac {r\hat{\epsilon}}{4}>0,\quad \forall t\in\tilde{U}+\operatorname{cl}(B+\hat {U}). $$

Thus,

2.25 $$ \hat{f}(y)< 0, \quad \forall y\in-\bigl(\tilde{U}+\operatorname{cl}(B+\hat {U}) \bigr). $$

This together with (2.23) leads to

$$\operatorname{cone}\bigl(A-\{\bar{y}\}\bigr)\cap\bigl[-\bigl(\tilde{U}+ \operatorname {cl}(B+\hat{U})\bigr)\bigr]=\emptyset. $$

8^∘^ Since *Ũ* is open, we have $\tilde {U}+\operatorname{cl}(B+\hat{U})$ is open, we get

2.26 $$ \operatorname{cl}\,\operatorname{cone}\bigl(A-\{\bar{y}\}\bigr)\cap\bigl[-\bigl( \tilde {U}+\operatorname{cl}(B+\hat{U})\bigr)\bigr]=\emptyset. $$

Since $\operatorname{cl}\,\operatorname{cone}(A-\{\bar{y}\})$ is a cone, we get

$$\operatorname{cl}\,\operatorname{cone}\bigl(A-\{\bar{y}\}\bigr)\cap\bigl[- \operatorname {cone}\bigl(\tilde{U}+\operatorname{cl}(B+\hat{U})\bigr)\bigr]=\{0 \}. $$

By [10], Definition 2.1, we have

$$\bar{y}\in HE\bigl(A,\operatorname{cl}(B+\hat{U})\bigr). $$

Since $\operatorname{cl}(B+\hat{U})$ is bounded, from [10], Proposition 3.2, we have

$$\bar{y}\in HE\bigl(A,C_{\hat{U}}(B)\bigr). $$

From [10], Proposition 3.5, we have

$$\bar{y}\in \operatorname{SE}\bigl(A,C_{\hat{U}}(B)\bigr). $$

The proof is complete. □

## Conclusions

In this paper, some properties for the interior of positive dual cones were studied. Using the dilating cones, we introduced a new notion of inner superefficient points, which has a nice property (see Theorem 2.3): suppose for each $y\in Y$, $A-\{y\}$ is nearly *C*-subconvexlike, and *B* is a bounded base of *C*, then $\bigcup_{U\in N(0)}\operatorname{SE}(A,C_{U}(B))=\operatorname{SE}(A,C)$. Hence it is really ‘approximate’. When the interior of *C* is empty, however, $\operatorname{int}C_{U}(B)\neq\emptyset$, in this case, we can obtain the properties of $\operatorname{SE}(A,C)$ by investigating $\operatorname{SE}(A,C_{U}(B))$. The research on the inner points of a set is very important in the study of multiobjective optimization. Hence, further research on the inner superefficient solutions of the set-valued optimization problem seems to be of interest and value.
